# Phenolic Composition of Brazilian BRS Carmem (Muscat Belly A × BRS Rúbea) Grapes: Evaluation of Their Potential Use as Bioingredients

**DOI:** 10.3390/foods12132608

**Published:** 2023-07-05

**Authors:** Yara Paula Nishiyama-Hortense, Carolina Olivati, José Pérez-Navarro, Reginaldo Teodoro Souza, Natália S. Janzantti, Roberto Da-Silva, Isidro Hermosín-Gutiérrez, Sergio Gómez-Alonso, Ellen Silva Lago-Vanzela

**Affiliations:** 1 Institute of Biosciences, Humanities and Exact Sciences (Ibilce), Campus São José do Rio Preto, São Paulo State University (Unesp), Rua Cristóvão Colombo 2265, São José do Rio Preto 15054-000, Brazil; yaranishy@gmail.com (Y.P.N.-H.); carolinaolivati@gmail.com (C.O.); natalia.soares-janzantti@unesp.br (N.S.J.); roberto.silva@unesp.br (R.D.-S.); 2 Instituto Regional de Investigación Científica Aplicada, Universidad de Castilla-La Mancha, 13071 Ciudad Real, Spain; jose.pnavarro@uclm.es (J.P.-N.); isidro.hermosin@uclm.es (I.H.-G.); 3 Brazilian Agricultural Research Corporation, Jales 15700-000, Brazil; reginaldo.souza@embrapa.br

**Keywords:** Brazilian hybrid grape, harvests, phenolic compounds, HPLC-DAD-ESI-MS^n^

## Abstract

The BRS Carmem grape was developed as an alternative for processing juices and wines. This study aimed to determine the phenolic compounds (PC) in the edible parts of this grape from two harvests—one harvested at ideal maturation time and another when the grapes were still immature—using HPLC-DAD-ESI-MS/MS. Student’s *t*-test was used (α = 0.05) to evaluate differences in the PC content between the edible parts and between the harvests. Both skins showed a predominance of flavonols, anthocyanins, hydroxycinnamic acids derivatives (HCAD) and stilbenes, with higher concentrations for harvest 1 than harvest 2. For both harvests (harvest 1 and harvest 2), the HCAD (mg of caftaric acid•kg fruit^−1^) was higher in whole grapes (383.98 and 67.09) than in their skins (173.95 and 21.74), with a predominance of *trans*-caffeic acid for all samples; the flavan-3-ols and proanthocyanidins (mg of (+)-catechin•kg fruit^−1^) presented higher concentrations in the seeds (flavan-3-ols: 203.20 and 182.71, proanthocyanidins: 453.57 and 299.86) than in the skins (flavan-3-ols: 1.90 and 4.56, proanthocyanidins: 37.58 and 98.92); the stilbenes concentration (µg 3-glc-resveratrol•kg fruit^−1^) was higher for the seeds from harvest 2 (896.25) than those from harvest 1 (48.67). BRS Carmem grapes contain a phenolic composition complex, and still have a relevant concentration of flavonols, anthocyanins and stilbenes, even when immature.

## 1. Introduction

To manage the loss and waste of fruits, it is important to seek their utilization as raw materials, even when they do not meet commercial standards for direct sale but are still suitable as a source of valuable compounds. This is a key strategy for achieving sustainable development and ending the rising hunger and food insecurity around the world [1,2]. As reported by the United Nations Food and Agriculture Organization (FAO) (2020) [1], over 60 million individuals in Brazil alone face some form of food insecurity and have limited access to healthy food options such as fruits and vegetables [3,4,5].

In this regard, fruits and vegetables are among the most highly perishable foods, and when marketed require a high standard of quality, either as a raw material for processing or for fresh consumption. Among the fruits, grapes are the most commonly grown worldwide due to their wide commercialization both as fresh and raw material for different products [6]. Viticulture is an important economic activity in Brazil, together with other countries in South America [7]. Brazil has also developdc new grape cultivars with differentiated productive cycles and high yields, as well as genetically improved physical-chemical characteristics, content of phenolic compounds (PC), and sensorial characteristics, such as flavor. This advance in the country’s viticulture should be disseminated to the rest of the world [8].

A grape’s taste and quality are closely related to the basic physical-chemical composition and to the secondary metabolites produced (phenolic compounds (PC), and volatiles) [9]. It is known that the maturation of a grape and consequently its phenolic composition are influenced by several biotic and abiotic factors, such as by the variety or cultivar, edaphoclimatic conditions of growth and the vegetative and reproductive development of the grapevine [6,10], through different viticultural practices such as the use of different rootstocks [11,12], the irrigation of vineyards [6] and the use of plant hormones or growth regulators [13,14].

The study of the impact of different variables on the phenolic profile of new hybrid grape cultivars grown annually with different soil and climate conditions and cultivation techniques is challenging, especially when compared to traditional cultivars in established wine regions with standardized growing conditions [15,16]. Some cultivars have resulted in fruit loss for producers due to difficulties in handling and meeting quality standards, affecting not only the local economy but also the most vulnerable people who could benefit from consuming the still edible fruits.

Several studies have highlighted the functional properties of PC present in grapes, particularly emphasizing their role in reducing the risk of non-communicable diseases, and showing that dietary intake of polyphenols derived from grape products can contribute to human health benefits [17,18].

Among the newly developed grape cultivars, BRS Carmem (Muscat Belly A × BRS Rúbea) stands out with its intense purple color, strong aroma, and raspberry-like flavor. Moreover, when harvested at the ideal maturation point (with a total solids content of approximately 19° Brix), it possesses favorable physical–chemical characteristics that make it suitable for the development of derived products. Previous research has investigated the PC content in BRS Carmem juice [11,19] and wine [20,21]. However, little is currently known about the PC content in the edible parts of both unripe and ripe BRS Carmem grapes, highlighting the need for further research in this area. Harvesting grapes at appropriate maturation stages is recognized as crucial for ensuring their quality as raw materials for processing. However, even in cases where early harvesting is necessary due to production cycle challenges, it is essential to explore alternative processes that allow for their utilization in the development of other value-added products.

Thus, the objective of this investigation was to assess the qualitative and quantitative aspects of PC (anthocyanins, flavonols, flavan-3-ols (monomers and dimers), proanthocyanidins (PA), hydroxycinnamic acid derivatives (HCAD) and stilbenes) in two distinct harvests of BRS Carmem grapes with different stages of maturation (one harvested at ideal maturation time and the other still unripe). The profiles of PC were determined via high-performance liquid chromatography using diode array detection coupled with electrospray ionization mass spectrometry (HPLC-UV-DAD-ESI-MS/MS). Due to their significant potential for supporting human health, it is crucial to acknowledge the bioactive components in fruits and to increase research into their utilization, even if the crop does not meet all market standards.

## 2. Materials and Methods

### 2.1. Chemicals

The solvents used were of chromatographic grade (>99%) and the chemical standards were of analytical grade (>95%). During the experiments, ultrapure water was used (Milli-Q system). The chemical standards malvidin (mv) 3-glucoside (3-glc), mv 3,5-diglucoside (3,5-glc), peonidin (pn) 3,5-glc, *p*-coumaric acid, *trans-*piceid acid, *trans-*caftaric acid, (-)-epigallocatechin (EGC) and (-)-gallocatechin (GC) were obtained from Phytolab (Vestenbergsgreuth, Germany). Cyanidin (cy) 3-glc, cy 3,5-diglc, procyanidins B1 (PB1) and B2 (PB2), kaempferol (K), quercetin (Q), isorhamnetin (I), myricetin (M), syringetin (S) and the 3-glc of K, Q, I and S and the 3-galactosides (gal) of M, K, Q and I, GC, EGC, (-)-catechin 3-gallate (CG) were obtained from Extrasynthese (Genay, France); meanwhile, *trans-*resveratrol (resv), caffeic acid, gallic acid, (+)-catechin (C), (-)-epicatechin (EC), (-)-epicatechin 3-gallate (ECG) and (-)-gallocatechin 3-gallate (GCG) were obtained from Sigma Aldrich (Tres Cantos, Madrid, Spain). The commercially unavailable standards procyanidin B4 (PB4), M 3-glc, Q 3-glcU and laricitrin (L) 3-glc had previously been isolated from Petit Verdot grape skins [22]. The *cis*-isomers of resv and its 3-glc (piceid) were obtained according to the methodology previously described [23].

### 2.2. Grapes

Two harvests of the BRS Carmem grape, one when ripe and the other when still unripe, were donated by the Experimental Station of Tropical Viticulture (Embrapa), located in the Northwest of São Paulo, Brazil, at 20°16′07″ South and 50°32′58″ West, and 500 m above sea level [24]. The first and second harvests were grafted, respectively, onto the ‘IAC-572 Jales’ (V. caribaea × 101-14 Mgt) and ‘IAC-766 Campinas’ (106-8 Mgt × Vitis caribaea DC.) rootstocks. According to Köppen’s classification, the climatic conditions of the region in which the two harvests were cultivated were: a climate classified as Aw (tropical climate with dry winter season), humid tropical, with concentrated rains from October to March [25], an average annual rainfall of 1449 mm, and an average annual temperature of 22 °C. The soil is classified as “Argissolo Vermelho-Amarelo” according to the Brazilian system of soil classification [26], which corresponds to an Ultisol [27]. Climate parameters were similar in both years and some observations regarding the variable factors of climate condition were referred throughout the text to in order to conduct reliable comparisons.

### 2.3. Physicochemical Characteristics

The physical–chemical characteristics (moisture, hydrogen potential (pH), total acidity (TA, as tartaric acid g•100 g^−1^), soluble solids (SS, °Brix at 25 °C) and SS/TA ratio of the fruit were determined, in triplicate, according to the Association of Official Analytical Chemists (2012) [28]. Moisture was determined gravimetrically and to carry out the analysis, samples of 5 g of grapes (randomly selected) were placed in porcelain crucibles previously weighed. They were put in an oven (315 SE, Fanem^®^,Guarulhos, Brazil) at 105 °C. Every three hours, the samples were weighed on an analytical balance (AY220, Shimadzu^®^ Barueri, Brazil) until reaching a constant weight. Then, the moisture results were obtained by the difference between the previous weight (wet) and the constant weight (dry). It was expressed as g water per 100 g of wet sample (%). For pH and total acidity analysis, 10 g of grapes (randomly selected) were homogenized (Heidolph DIAX 900, Merck, Kelheim, Germany) with 100 mL of Mili-Q water. Then, the pH was determined using a pH meter (Tecnal, TEC-5) and the total acidity was measured via titrimetry using NaOH 0.1 M (Dinâmica, Indaiatuba, Brazil) and phenolphthalein as a pH indicator. The SS determination from the grapes was performed by means of direct reading in an Abbe Refractometer (Quimis, Q767B, Diadema, Brazil).

In addition, the average mass (g), length (L, mm), width (W, mm) of the grapes were determined. Ten grapes (randomly selected) were weighed on an analytical balance (AY220, Shimadzu^®®^) to obtain the berry mass, and then measurements of length and width were taken to obtain the berry size (L × W) with the aid of a Universal Pachymeter (150 mm, Inox, 0.02 mm, Digimess, São Paulo, Brazil).

### 2.4. HPLC-DAD-ESI-MS^2^ Identification and Quantification of Phenolic Compounds of Grape Parts

Detailed determination of PC (anthocyanins, flavonols, HCAD, stilbenes, flavan-3-ols (monomers and dimers), and PA) were conducted using previously described methods [23]. To avoid oxidation problems, anthocyanins’s determination was done only with the whole grape (WG), as it is a non-teinturier grape. Flavonol and HCAD were performed with the WG and grape skin (SK). Flavan-3-ols, PA and stilbenes analyses used skin and seeds. For WG and skins of BRS Carmem grape of the harvest 1 (denominated WG1 and SK1, respectively) and harvest 2 (WG2 and SK2, respectively), the samples (*n* = 2; ca. 100 g per sample) were subjected to three repeat extractions for recovery of the PC according to the methodology described by Rebello et al. (2013) [23] using a solvent mixture of methanol, water, and formic acid (70:28.5:1.5, *v*/*v*). Seeds from harvest 1 (SE1) and harvest 2 (SE2) (ca. 2 g) were crushed and homogenized (Heidolph DIAX 900, Merck, USA) with 50 mL of the extracting solution containing methanol, water, and formic acid (50:48.5:1.5 *v/v*). All of the supernatants recovered from the three extractions were combined and dried in a rotary evaporator (35 °C), their volume was made up to 100 mL with water, then they were stored at −18 °C (Ultra-freezer Sanyo MDF-U56VC, Panasonic, Osaka, Japan) [23]. All extractions were realized in triplicate.

For anthocyanins analysis, aliquots (5 mL) from extracts of the WG were submitted to an extraction using SPE-C18 cartridges (Waters, Milford, USA) to remove the sugars and others polar compounds as described by Olivati et al. (2022) [8] and injected (10 µL) into the chromatographic column. Prior to the analysis of flavonols and HCAD analysis, to obtain an anthocyanin-free and sugars-free fraction, aliquots (3 mL) from prepared samples of WG and skins were extracted in Bond Elut Plexa PCX cartridges (Agilent Technologies, Santa Clara, CA, United States) [29] and injected (20 μL) into the chromatographic column.

The HPLC separation, identification and quantification of anthocyanins, flavonols and HCAD were carried out using the same conditions as described by Rebello et al. (2013) [23] on an Agilent 1100 Series system (Agilent Technologies, Waldbronn, Germany), equipped with DAD (G1315B) and a LC/MSD Trap VL (G2445C VL) electrospray ionization mass spectrometry (ESI-MS^n^) system, and coupled with an Agilent Chem Station (version B.01.03) data-processing station. The mass spectra data were processed with the Agilent LC/MS Trap software (version 5.3). For quantification, DAD-chromatograms were extracted at 520 (anthocyanins), 360 (flavonols) and 320 nm (HCAD). The usual information of MS and MS/MS spectra (*m*/*z*) for these compounds was used, and the retention times found are described in Table S1.

The HPLC separation, identification and quantification of flavan-3-ol, PA and stilbenes from the skins and seeds were carried out using an HPLC Agilent 1200-series system coupled with an AB Sciex 3200 QTRAP (Applied Biosystems, Foster City, CA, USA) with triple quadrupole, turbo spray ionization mass spectroscopy system (ESI-MS/MS) operating in Multiple Reaction Monitoring (MRM) mode. The chromatographic system was managed using an Agilent ChemStation (version B.01.03) data-processing unit, and the mass spectral data were processed using the Analyst MSD software, version 1.5 (Applied Biosystems, Foster City, CA, USA) as described by Olivati et al. (2022) [8]. For identification, a previously developed method based on the use of the EMS (enhanced mass spectrum; MS conditions) scan mode was used, as described by Colombo et al. (2011) [29], with the MRM (multiple reaction monitoring; MS/MS conditions) scan mode being used for quantification, (+)-catechin used as an external standard, and acid-catalyzed depolymerization induced by pyrogallol used for the structural characterization of proanthocyanidins. The identification and quantification of stilbenes (*trans* and *cis* isomers of resveratrol and piceid) were performed based on the extracted ion chromatograms obtained by MRM after selection of the following characteristic *m*/*z* transitions: 389–227 for piceid isomers; and 227–185 for resveratrol isomers [8,23].

All the standards were used for identification and quantitation through calibration curves covering the expected concentration ranges. Flavonols and anthocyanins identified in the samples were presented quantitatively in the form of molar ratio (%), normalized to the total content. The sum of all compounds of the same type was quantitatively reported as the total concentration, with results expressed, respectively, as mg equivalents of quercetin 3-glucoside (Q-3-glc) for the flavonol 3-glycosides, malvidin 3-glucoside (mv-3-glc) for anthocyanidin 3-glucosides and malvidin 3,5-diglucoside (mv-3,5-diglc) for anthocyanidin 3,5-diglucosides.

For non-available standards, the quantitation was performed as equivalents of the most representative compounds for each family of phenolic compounds: caftaric acid for the HCAD; (+)-catechin for polymeric flavan-3-ols (total PA); and individual flavan-3-ol monomers and dimers by their corresponding standards, but their total sum as (+)-catechin equivalents. In the specific case of the stilbenes, as well as that of flavan-3-ols monomers and dimers (B-type procyanidins), the molar ratio of the compounds was not presented. The stilbenes were quantified directly with their respective standards (*trans* and *cis* isomers of resveratrol and its 3-glucoside (piceid)). For the identification and quantification of diverse flavan-3-ols, we used standards of: the monomers (+)-catechin, (−)-epicatechin, (−)-epigallocatechin, (−)-gallocatechin, and (−)-epicatechin 3-gallate, and the dimers procyanidins B1, B2 and B4, expressing their total sum as mg of (+)-catechin equivalents. The total polymeric PA content was quantified as equivalents of (+)-catechin, and their structural features were characterized (molar percentage of extension and terminal subunits; mean degree of polymerization, mDP; molar percentage of galloylation; and molar percentage of prodelphinidins).

### 2.5. Data Analysis

To compare two means between the edible parts and also between harvests from BRS Carmem, Student’s *t*-test was used at a significance level of 0.05 (α = 0.05). All the statistical analyses were carried out using the SPSS statistical software (SPSS, IBM, V20).

## 3. Results

### 3.1. Physicochemical Characteristics

Based on the results for physicochemical characteristics (Table 1) of the ripe grapes (harvest 1) and unripe grapes (harvest 2), the grapes had moisture, TA, SS and SS/TA values which were significantly different (*p* ≤ 0.05) from each other. The moisture value is within the range reported for other grapes, such as for BRS Violeta (76%) [30]. To compare the results obtained for TA, SS and SS/TA with those in the literature, we can cite the study conducted by Tecchio et al. (2022) [16], in which the physicochemical characteristics of BRS Carmem grafted on ‘IAC-572 Jales’ and ‘IAC-766 Campinas’ rootstocks were investigated. In that study, the mean values for TA (0.97 g tartaric acid•100 g grape^−1^), SS (17.6 °Brix), and SS/TA (18.8) are within the range found in our study [31].

Cheng et al. (2017) [33], after evaluating the production of Red Alexandria grapes with 15 rootstocks, reported that the variation of the rootstock can cause a different accumulation of sugars in the grapes produced by the graft cultivar. The authors reported that the greater the vigor of the rootstock, the greater the leaf density of the descendant. Under these conditions, there may be a favorable effect on the capture of solar energy, consequently leading to an improvement in the photosynthetic efficiency of the vine. Consequently, higher carbohydrate contents would be available to be transported to the grapes. Silva et al. (2018) [11] also evaluated the BRS Carmem grape with ‘IAC-766′ and ‘IAC-572′ rootstocks and reported SS values of 15.1 and 14.8 °Brix, respectively. Borges et al. (2014) [34], after evaluating six clones of the Concord grape with ‘IAC-572′ and ‘IAC-766′ rootstocks, obtained SS values ranging from 11.4 to 13.7 for the ‘IAC-572′ and from 11.6 to 13.2 for the ‘IAC-766′. Brito et al. (2019) [35], on the other hand, after determining the physicochemical characteristics of Arizul grapes from two harvests with ‘IAC-572′, ‘IAC-313′, ‘IAC-766′ and ‘Paulsen-1103′ rootstocks, reported that the highest SS values were obtained for the grapes produced with the rootstock ‘Paulsen-1103′, which is the least vigorous rootstock among those tested. These authors also determined the physicochemical characteristics for BRS Clara grapes with the same rootstocks and reported that the moderately vigorous rootstock (‘IAC-313′) was the one with the best results regarding SS, even though these values did not differ from those obtained with ‘IAC-766′ and ‘Paulsen-1103′ rootstocks [35].

The pH values of grapes are close to those reported in the Technical Release provided by Embrapa (3.6) [31], as well as those reported for red BRS Magna grapes (3.7) [36] and for BRS Violeta grapes (3.8) [37]. Nevertheless, Tecchio et al. [16] investigated BRS Carmem grapes and found a lower pH value (3.2). De-Assis et al. (2005) [38], when evaluating the physicochemical characteristics of BRS Carmem grapes produced in northern Paraná, reported an SS value of 13.7, TA (g of tartaric acid•100 g of grape^−1^) of 0.90, pH of 3.17 and SS/AT of 15.7.

Brito et al. (2019) [35], after determining the physicochemical characteristics of BRS Clara grapes, observed marked differences in relation to the two productive cycles analyzed, which resulted among all rootstocks in mean SS/TA values of 54.6 and 23.6. This index helps in deciding the ideal moment for harvest; because SS and TA have an inverse evolution relationship, they tend to be similar in relation to the evolution of SS, i.e., there is a progressive increase in SS/TA values until the period near the harvest [39].

The balance between TA and SS is extremely important for grape’s flavor quality, and producers often make changes in the production cycle so that they can harvest during drier months, aiming to increase the levels of SS [40]. The higher this value, the better the quality of the raw material.

Based on the obtained results and after comparison with the literature, it can be verified that depending on the edaphoclimatic conditions of cultivation, as well as the type of rootstock utilized and the vine planting and management practices, the productive cycles of this cultivar may exhibit certain distinct physicochemical characteristics primarily due to the lack of adherence to the ideal maturation stage. However, there are studies in the literature pointing to immature grapes as important food resources, such as as raw material for drinks production with reduced sugar and alcohol content [41]; an alternative in sauces substituting vinegar or lemon juice [42]; antioxidant compounds for use as an anti-browning agent in white wines [43]; and as a source of PC [44]. Thus, immature grapes, such as in the second harvest analyzed in this study, still deserve attention and investigation for their potential related to compounds with bioactive properties.

### 3.2. Qualitative and Quantitative Determination of PC by HPLC-DAD-ESI-MS^2^

#### 3.2.1. Flavonols and Anthocyanins

The flavonols detected and identified in both harvests (Table 2) were derivatives of the Q, I, M, L and S aglycones. No flavonol derived from K was found, which is the first compound formed in the biosynthetic route of flavonols and soon transformed into other compounds [22]. This compound was also absent in the hybrid BRS Violeta [23] and the *Vitis vinifera* Italia [45] grapes. Free flavonols were reported in harvest 1, and this suggested that during the extraction process of the grapes, acid hydrolysis of these compounds might have occurred. Therefore, to compare the results of the individual molar ratios of flavonols obtained for each harvest, the molar proportions were recalculated per type of flavonol aglycone (M-type, Q-type, L-type, I-type and S-type).

Both harvests (Table 2) showed higher concentrations of M-type flavonols, with 81% and 76%, respectively for WG1 and SK1, and 51% and 48%, respectively for WG2 and SK2, followed by flavonols of Q-type flavonols. In addition to the free form, for harvest 1, flavonols linked to a glucuronide (3-glcU), galactoside (3-gal) and glycoside (3-glc) were identified. A similar flavonol profile to that determined for BRS Carmem harvest 1 was described for the Bordô grape, in SK and flesh analyzed separately; however, the flavonol K-3-glc was present in Bordô grapes [46], but not in BRS Carmem (in the present study). The Rufete (*Vitis vinifera*) [47] and BRS Violeta grapes [23] also showed a similar flavonol profile to BRS Carmem in the present study, but with the absence of flavonols from I and the presence of flavonols from K. The coumaroylated flavonol derived from S (S-cmglc), recently found and identified by Favre et al. (2018) [48] in *Vitis vinifera* grapes and their wines, was identified in the present study for BRS Carmem of harvest 2 with concentrations of 0.8% for WG2 and 0.8% for SK2.

Liang et al. [49] evaluated the commercial developmental patterns of the individual flavonols from three grape cultivars grown in the same region during two consecutive years and found that the edaphoclimatic conditions, in particular the solar incidence, rainfall volume during the production cycle and temperature between the annual harvests markedly influenced the flavonol profile of the grapes. The authors reported, for example, that the flavonols Q-3-glc and Q-3-gal were not detected in Merlot and Cabernet Gernischt from the first harvest, whereas they were present in the following harvest. Moreover, some cultivars were more phenologically and climatologically sensitive, which can result in a variation in qualitative and quantitative composition in the same cultivar from one season to another.

Analyzing the qualitative profile of anthocyanins for BRS Carmem, we found 26 compounds for WG1 and 31 compounds for WG2, which were derived from the five main anthocyanidins; delphinidin (dp), cy, pn, petunidin (pt) and mv were found, while diglycosylated (3,5-glc) and monoglycosylated (3-glc) derivatives in both non-acylated and acylated (acetylated, cumarylated, and some caffeylated) forms were also found (Table 3). The major anthocyanins were derived from dp for WG1. However, for WG2, the major anthocyanins were derivates from mv. The *p*-coumaryl monoglycosylated (3-cmglc) derivatives from pt, pn and mv were identified for both harvests, but the derivative from cy was present just in WG2. Acetylated diglycosydes (3-acglc-5-glc) derived from pn and mv were detected in both harvest, but in addition, the derivative of pt was identified for WG2. The acetylated monoglycosylated (3-acglc) derivatives from dp, pt and mv were reported for both harvests, and the derivative from pn was also found for WG2. The *p*-caffeylated anthocyanins were found in monoglycosylated form (3-cfglc) only for WG2 and from mv, as well as the *p*-caffeylated diglycosyde anthocyanin (3-cfglc-5-glc) derivatives of cy, pt, pn and mv. On the other hand, for WG1, there was only the *p*-caffeylated diglycosyde derivative from dp.

For WG1, the anthocyanins derived from dp were also predominant for BRS Violeta [23]. The predominance of anthocyanins derivatives from mv, as reported for WG2, was also noted for Bordô grape skins [46] and for the hybrid grape Maximo [50]. Higher concentrations of mv derivatives were also reported by Silva et al. [11] for BRS Carmem skins grown on the same rootstocks use in the present study. These authors reported the presence of mv-3,5-glc, cy-3,5-glc and 3-glc derived from mv, cy, dp, pn and pelargonidin anthocyanins. For BRS Vitória grapes, the authors reported that in the monoglycosylated anthocyanin fraction, there is a higher predominance of the derivatives from dp, while for the diglycosylated anthocyanin fraction, there is a predominance of mv derivatives [29]. From the results of our study, it can be established that the predominance of mv or dp among the anthocyanins of BRS Carmem grape was influenced not only by the grape cultivar.

Both monoglycosylated and diglycosylated anthocyanins are seen, although the relationship between these two forms (ratio 3,5glc/3glc; 3,5glc, diglycosylated and 3glc, monoglycosylated) showed that there is a predominance of diglycosylated anthocyanins for both harvests, with values significantly higher for WG2 (5.6) than WG1 (2.2). In this context, BRS Carmem grape presented percentages of diglycosylated anthocyanins for WG1 of 66% and for WG2 of 85%, and these results are in accordance with the genealogy of this cultivar [31].

There are reports in the literature of non-*vinifera* grapes with a percentage of diglycosylated anthocyanins of approximately 90%, such as for Bordô grapes [46] and BRS Violeta grapes [23]. It is noteworthy that the lower the ratio, the smaller the difference between the concentrations of monoglycosylated and diglycosylated anthocyanins.

In the present study on BRS Carmem grapes, the qualitative profiles of the flavonols and anthocyanins showed that, for both harvests, there was a predominance of tri-substituted flavonols, with values of 90% (WG1), 89% (SK1), 75% (WG2) and 70% (SK2), as well as of tri-substituted anthocyanins (dp, pt and mv), both in the monoglycosylated fraction (92%) and the diglycosylated fraction (92% and 94%), respectively, for WG1 and WG2. WG1 showed a higher concentration of non-acylated diglycosylated anthocyanins (57%) than monoglycosylated anthocyanins, which were mostly acylated (54%); meanwhile, WG2 presented a higher concentration of acylated anthocyanins, both monoglycosylated (82%) and diglycosylated (85%). Silva et al. (2019) [19], studying the influence of different rootstocks, reported that the proportion of di-substituted anthocyanins to tri-substituted anthocyanins for Cabernet Sauvignon grapes was also affected by the differences in the rootstocks.

It can be suggested that for both harvests, during grape development, a higher activity of the F3’5’H enzyme in the biosynthetic pathway of flavonols and anthocyanins occurred. That is because a higher concentration of tri-substituted compounds was observed, especially of M-type flavonols and the anthocyanins dp-3-glc, mv-3-glc and pt-3-glc. According to the general route of this biosynthetic pathway, an expressive activity of the enzyme 5-*O*-glycosil transferase probably resulted in the formation of diglycosylated anthocyanins derived from dp, mv and pt. It is still not clear how the complex interactions between abiotic and biotic factors influence the activity of acyltransferase enzymes involved in the formation of acylated anthocyanins [49]. However, the results of the present study suggest that the growing conditions of the first harvest positively enabled the anthocyanin acylation. This is a potential advantage for these grapes due to the greater color stability of acylated anthocyanins when compared to its corresponding non-acylated anthocyanins [51].

Regarding the quantitative profile of flavonols (mg Q-3-glc•kg fruit^−1^, Table 2), harvest 2 showed significant differences between the edible parts (76.8 and 38.7 for WG2 and SK2, respectively), but this was not the case not for harvest 1 (70.5 and 68.5 for WG1 and SK1, respectively). All the samples showed lower values than reported for BRS Violeta skins (153 mg) [23] and higher than Bordô grape (approximately 154 µmol) [45]. In addition, the total concentration of flavonols present in SK1 represented approximately 97% of the fruit, whereas the concentration present in SK2 represents 51% of the fruit, which means that flavonols accumulated mainly in the skins of BRS Carmem grapes.

The concentration of flavonols in grapes remains relatively constant throughout the development of the berry [52]. Nevertheless, Silva et al. (2018) [11] showed differences in flavonols’ total concentration for BRS Carmem skins produced in harvest 1 (11.6) and harvest 2 (7.83), due, among other things, to differences in the chemical properties of the grapes, such as pH, TA and SS.

For the quantitative anthocyanin profile (Table 3), harvest 1 provides a significantly higher concentration of monoglycosylated anthocyanins than that of harvest 2, and the concentrations of diglycosylated anthocyanins were significantly different from each other. For the quantitative anthocyanin profile (Table 3), harvest 1 (559 mg mv-3-glc•kg fruit^−1^) provides a significantly higher concentration of monoglycosylated anthocyanins than that of harvest 2 (194 mg mv-3-glc•kg fruit^−1^), and the concentration of diglycosylated anthocyanins was similar between the two harvests (1650 and 1631 mg mv-3,5-diglc•kg fruit^−1^). Silva et al. [11] reported for BRS Carmem SK produced on ‘IAC-766′ rootstock a value of 2342 mg mv-3-glc•kg fruit^−1^, and Lago-Vanzela et al. (2011) [46] reported for Bordô grape skins a value of 1360 mg mv-3,5-glc•kg fruit^−1^.

The accumulation of anthocyanins in the skin of the grape berries occurs first due to the slower accumulation of anthocyanins, followed by a rapid increase and a stabilization phase. At the end of the ripening process, it is possible to observe a decrease in the concentration of this dye [52].

The difference between the total concentrations of anthocyanin (mg mv-3,5-glc•kg fruit^−1^) determined in harvests 1 (2528) and 2 (1921) may be related to the different degrees of maturation. The values found for both harvests were close for BRS Carmem SK produced on IAC-766 rootstock (2342 mg cy-3-glc•kg fruit^−1^) [11], as well as that reported for Bordô skins (1359 mg mv-3,5-glc•kg fruit^−1^) [23], despite the differences in the anthocyanins used to express the results. Previously, Oliveira et al. (2019) [53] found, for Syrah grapes, maximum anthocyanin values (g mv-3-glc•kg fruit^−1^) of 32.9 for a grape harvested in Bahia (Brazil) and 15.8 for a grape harvested in Pernambuco (Brazil), both lower than those reported in this study.

The interest in anthocyanins, besides their coloring properties, is also related to their potential health benefits, such as antioxidant action and protection against neural and cardiovascular diseases, cancers, diabetes, and inflammation, among others [54,55]. Flavonols are related to reducing the risk of developing pathogenic diseases, such as cancers [56] and cardiovascular diseases [57]. Several studies have investigated how to obtain such products (bioingredients and dyes) in solid (powder) and liquid forms or in an extracted form from fruits and vegetables, such as blueberry [58], *jabuticaba* [59], grape [60], purple carrot [61] and eggplant peel [62].

Flavonols, in addition to possibly having functional properties, can act as copigments, stabilizing anthocyanins through intermolecular copigmentation reactions and inhibiting anthocyanins’ degradation and prolonging color stability [63]. When comparing the concentrations of anthocyanins and flavonols in BRS Carmem grapes with those available in the literature, it is noticeable that even when immature, this cultivar is still rich in these compounds, and may be used as an alternative source for the production of bioingredients, either dehydrated or as extracted anthocyanins, to be used as natural dyes.

#### 3.2.2. Qualitative and Quantitative Profiles of Stilbenes, Flavan-3-ol Monomers and Dimers and Proanthocyanidins

The quantification of the flavan-3-ol monomers and dimers is shown individually and expressed in mg of each compound•kg fruit^−1^ (Table 4). The total concentration (sum of flavan-3-ol monomers and dimers) was also separated into concentrations of monomers and dimers. The total concentration of PA was expressed in mg of C•kg fruit^−1^; the PA structural characteristics such as %galoylation, %prodelfinidines, and the percentages of flavan-3-ols with extension units and terminal units were given in percentages (Table 4).

C, EC, GC, EGC and ECG were present in the SK1 sample, while their respective SE1 also contained CG in addition to these compounds. For harvest 2, the seeds presented C, EC, CG, ECG and two glycosylated monomers (MG1 and MG2), while in their respective skins, C, EC, GC, EGC and ECG were found. In BRS Carmem, the C was the major flavan-3-ol monomer and the seeds showed higher total concentration than the skins. These results were significantly higher for SE1 (179 mg C•kg fruit^−1^) than for SE2 (127 mg C•kg fruit^−1^). There was no significant difference between the skins (1.17 and 1.04 mg C•kg fruit^−1^ for SK1 and SK2, respectively).

For flavan-3-ol dimers, all samples presented the three type B dimers (PB1, PB2 and PB4); PB4 was predominant for both samples of SE1 and SK1, and for harvest 2, PB2 was the compound with the highest concentration for SE2, while PB1 was the highest for SK2. The total concentration of flavan-3-ols (TCF) dimers, within the same harvest, was higher in seeds than in skins, whereas harvest 2 showed higher concentrations than those of harvest 1, both for the seeds and for the skins. The TCF monomers dimers and sum were also significantly higher in the seeds than the skins. There were significant differences between the total concentrations of the sum of flavan-3-ols of the skins, with higher concentrations for harvest 2 than harvest 1.

The total concentration of PA was significantly higher in the seeds. However, in this case, the harvest 1 seeds showed a higher concentration, while in relation to the skins, harvest 2 showed a higher concentration than harvest 1. As expected, the % galloylation was also higher in the seeds, with values of 9 and 7%, respectively, for SE1 and SE2, as opposed to values of 2 and 4%, respectively, for SK1 and SK2, with only the skins showing a significant difference between the harvests. The skins had higher % prodelphinidins than the seeds, but, in this cultivar, these percentages were significantly higher in harvest 1. As usually found in grapes, the mDP was statistically higher in skins than in seeds.

BRS Violeta grape skins reported a higher proportion of C (49%) than the flesh (83%) and seeds (87%), and a TCF with values of 346 mg C•kg fruit^−1^ in the seeds and 8.6 mg C•kg fruit^−1^ in the skins [23]. Those values of TCF were higher than the reported for BRS Carmem grape.

The recorded mDP values in the skin samples of this study exhibited lower measurements in comparison to the findings reported by Lago-Vanzela et al. (2011) [45] for Bordô grape skins (12), and the %galloylation found in the skins of the grapes in the present study was lower than that for BRS Violeta SK (3%), except for the SK2 sample, which had a value of 4% (still quite near to 3%). For the seeds, the values found for PA and mDP in the present study were lower than those reported for the seeds of the BRS Violeta grape (12%) [23].

The percentage of prodelphinidins in all of the samples of skins in the present study was higher than that reported for the Bordô grape, which had an approximate value of 14.2% in its skins [46]. However, all of these values were lower than that reported for BRS Violeta skins (58%); similarly, in the same work, the authors report a value of 2% for BRS Violeta seeds [23], higher than found here. Regarding the structural characterization of PA, for terminal units, “% C-term” and “% EC-term” showed higher percentages for all samples. For extension units, “% EC-ext” occupied a higher percentage in both harvests, with a higher concentration in the seeds.

The different phenolic compositions between the harvests are supported by other studies. Rajha et al. (2017) [64] reported that the maximum concentration of polyphenols in the grapes was induced by higher temperatures (up to 25 °C) and lower rainfall. In addition, they described that thermal and water stresses have also been shown to increase polyphenolic production. When comparing the productive cycles of the two harvests studied, it can be highlighted that in the second harvest, the stages of veraison and complete maturation occurred with higher temperature values, and lower minimum humidity values. These facts may have enhanced the concentration of flavan-3-ol and proanthocyanidins in skin and seeds of BRS Carmem from the second harvest, even with incomplete maturation. These results show that the immature BRS Carmem grape, produced under these stress conditions, can be a relevant source of such compounds.

#### 3.2.3. Qualitative and Quantitative Profiles of Hydroxycinnamic Acid Derivatives and Stilbenes

For both harvests, qualitative profiles and the total concentration of HCAD are shown in Table 5. *Trans*-caftaric acid, *trans*-coutaric acid and *trans*-fertaric acid were identified, as well as the isomers of glycosylated coumaric acid called 1”-glucoside-coumaric ester, 2”-glucoside-coumaric ester and 3”-glucoside-coumaric esters. These esters and hexoses of hydroxycinnamic acid have been reported for other grape cultivars that are cultivated and imported for the Brazilian market, such as BRS Violeta grape [23] and Bordô grape [46]. The predominant HCAD for BRS Carmem was mostly *trans*-caffeic acid for all edible parts and harvests analyzed. This acid was also the major compound for the Isabel grape, followed only by *trans*-coutaric and *cis*-coutaric acids [65].

For harvest 1 and harvest 2, the second major compound was *trans*-coutaric acid and 3-glc-coumaric acid, respectively. There are reports that indicated that the HCAD profile may vary for the same grape cultivar, as was the case for BRS Violeta grape, which had variance in the HCAD profile: in most studies, *trans*-caftaric acid was the major compound, such as for BRS Violeta and BRS Lorena grapes produced in São Roque (Brazil) in 2011 and 2012 [66], but in this same study, the major compound in the skin was *p*-1-glc-cumaric acid; a 2013 harvest, produced in Jales (Brazil), showed a higher concentration of *trans*-caftaric acid in the whole fruit, followed by the fertaric acid [30].

In the present study, the HCAD total concentration (all expressed in mg caftaric acid•kg fruit^−1^) was significantly higher in WG when compared with their respective skins (Table 5). It was expected that these compounds accumulate at the skins and flesh [23]. For harvest 1, the concentration of these compounds in the skins represented 45% of the total, and for harvest 2, the concentration was 32% of the total. For WG, only WG1 showed a higher HCAD concentration than that reported for BRS Violeta grape (134 mg, the sum of skin and flesh) [23], for Bordô grape (483 µmol) [45] and also for Garnacha Tintorera (689–799 µmol) [22]; converting the results from WG1 to µmol, we obtain a value of 1999 µmol. The HCAD results obtained for all of the examined skin samples in this investigation demonstrated higher values compared to those observed for BRS Violeta skins (14 mg) [23].

Silva et al. (2018) [11] also studied BRS Carmem grapes and did not report any significant differences between the results for the phenolic acids present. Costa et al. [67], studying the influence of rootstocks in two distinct productive cycles of the Chenin Blanc grape, verified that the same rootstock can present different results between the cycles. In the present study, harvest 1 provided a higher concentration of HCAD. A similar result was disclosed for one of the productive cycles of the Chenin Blanc grape [67]. In view of these results, it is suggested that harvest 1 provided a greater quantity of 4CL and C3H enzymes [68] when compared to harvest 2 for BRS Carmem grape.

The stilbene profile and the total concentration (3-glc-resv•kg fruit^−1^) are shown in Table 6. The *cis*- and *trans*-piceid isomers were identified in both harvests. The *cis*-resv compound was only identified in harvest 2. Stilbenes were seen in higher concentrations in the seeds than in the skins. The total concentrations of stilbenes for harvest 1 were 48.7 µg and 21.8 µg for seeds and skins, respectively, and for harvest 2 they were 896 µg and 3.69 µg for seeds and skins, respectively. For Bordô grapes, higher values were reported than for the samples studied here, at 10.9 mg [46].

The results suggest that harvest 1 induced a higher concentration of piceid derivatives than harvest 2, probably due to the higher activity of the GT enzyme. On the other hand, harvest 2 significantly induced the formation and concentration of resv derivatives, probably due to greater activity of the STS enzyme [69], which may be further investigated in future studies.

## 4. Conclusions

It was possible to determine the phenolic characterization (flavonols, anthocyanins, flavan-3-ols, proanthocyanidins, hydroxycinnamic acids and stilbenes) of the edible parts (whole grape, skin, and seeds) of the BRS Carmem grape, as well to expand the knowledge about the composition of this cultivar in both mature and immature stages. In the grape skins, there is a predominance of flavonols, anthocyanins, HCAD and stilbenes, and the ripe grape (harvest 1) showed higher concentrations than the unripe grape (harvest 2). For both harvests, the qualitative profiles of the flavonols showed that there was a predominance of tri-substituted compounds, while for the anthocyanins in monoglycosylated and diglycosylated fractions, there were a prevalence of tri-substituted compounds. The M-type flavonol was the dominant compound in all samples analyzed. As for the anthocyanins, the derivatives from dp were the major compounds in harvest 1, while the derivatives from mv were the major compounds in harvest 2. The HCAD total concentration was higher in WG than in their respective skins. For all edible parts and harvests, *trans-*caffeic acid was the predominant HCAD. For stilbenes profile, *cis-* and *trans-*piceid isomers were identified in both harvests. It should be noted that for WG, the total concentrations of flavonols did not differ between the harvests, and S-cmglc, the recently reported flavonol for grapes, was present only for harvest 2. Furthermore, in harvest 2, there was a high concentration of stilbenes in the seeds, which suggests the potential for further exploration in the development of new studies. For both harvests, flavan-3-ols (monomers and dimers) and PA were found in higher concentrations in the seeds than in their respective skins. In addition, there were higher total concentrations of flavan-3-ol and PA in the seeds for the harvest 1 than for harvest 2. All samples exhibited a high concentration of the compound C for fla-van-3-ol monomers. In the case of harvest 2, two MG monomers were detected in the seed samples. For flavan-3-ol dimers, PB4 was the predominant compound in all samples from harvest 1. However, in harvest 2, PB2 was the predominant compound in the seeds, while PB1 was the predominant compound in the skins.

In summary, the results indicate that both ripe and immature grapes contain significant levels of various classes of PC, making this cultivar suitable as a raw material for developing products or bioingredients such as hydroalcoholic extracts or powdered preparations for distinct food products. This can contribute to a food system that promotes human well-being, social equity, and the realization of a “circular society.”

## Figures and Tables

**Table 1:** Physicochemical characteristics, berry size and berry mass of the ripe (harvest 1) and unripe (harvest 2) BRS Carmem grapes (*n* = 3).

	Harvest 1	Harvest 2
Moisture (%)	74.4 ± 0.16	81.50 ± 0.46
pH	3.56 ± 0.29	3.75 ± 0.04
TA (tartaric acid g•100 g^−1^)	0.82 ± 0.10	1.16 ± 0.16
SS (°Brix at 25 °C)	19.50 ± 0.00	12.97 ± 0.06
SS/TA ratio	23.93 ± 2.82	11.34 ± 1.43
L × W (mm × mm)	15.5 × 13.6	17.6 × 15.6
Berry mass (g)	2.28 ± 0.31	2.36 ± 0.23

pH, hydrogen potential; TA, total acidity; SS, soluble solids; L, length; W, width. Adapted from Nishiyama, 2020 [32].

**Table 2:** Flavonols in whole grapes and skins of BRS Carmem grape according to HPLC-DAD-ESI-MS^2^ (negative ionization mode), molar profiles (percentage of each individual flavonol and flavonol aglycones types regarding the total content) and total concentration given as mean values ± standard deviation (*n* = 2).

Flavonol ¹	% Molar Percentages			
	WG1	SK1	WG2	SK2
M-glcU	4.98 ± 0.55aB	4.60 ± 0.67aB	9.07 ± 0.34aA	9.72 ± 0.61aA
M-gal	2.39 ± 0.36a	1.29 ± 0.01a	ND	ND
M-glc	72.23 ± 0.62aA	56.97 ± 6.78aA	41.96 ± 0.31aB	38.40 ± 3.79aA
Q-gal	ND	ND	0.99 ± 0.08a	0.68 ± 0.62a
Q-glcU	5.55 ± 0.68aA	4.70 ± 1.77aB	10.68 ± 1.72aA	15.05 ± 0.14aA
Q-glc	4.84 ± 0.10aB	3.93 ± 1.44aB	10.63 ± 1.61aA	11.71 ± 2.33aA
L-glc	6.33 ± 0.46bB	9.73 ± 0.99aB	16.14 ± 2.10aA	14.05 ± 0.19aA
I-glc	0.10 ± 0.03aB	0.1 0.88aB	2.25 ± 0.07aA	2.14 ± 0.35aA
S-gal	0.42 ± 0.05a	0.24 ± 0.05a	ND	ND
S-glc	1.71 ± 0.18aB	1.74 ± 0.54aB	7.44 ± 1.33aA	7.48 ± 0.60aA
S-cmglc	ND	ND	0.83 ± 0.08a	0.76 ± 0.17a
M free	1.44 ± 0.12b	12.63 ± 0.80a	ND	
Q free	ND	1.78 ± 0.85	ND	ND
L free	ND	0.57 ± 0.20	ND	ND
I free	ND	0.43 ± 0.38	ND	ND
S free	ND	1.28 ± 0.73	ND	ND
M-type	81.04 ± 1.40aA	75.49 ± 6.92aA	51.03 ± 0.03aB	48.12 ± 4.41aB
Q-type	10.39 ± 0.78aB	10.40 ± 4.06aB	22.31 ± 3.41aA	27.45 ± 3.09aA
L-type	6.33 ± 0.46bB	10.30 ± 1.19aB	16.14 ± 2.10aA	14.05 ± 0.19aA
I-type	0.10 ± 0.03aB	0.54 ± 0.46aA	2.25 ± 0.07aA	2.14 ± 0.35aA
S-type	2.14 ± 0.13aB	3.27 ± 1.21aB	8.27 ± 0.41aA	8.25 ± 0.78aA
Total (mg Q-3-glc/kg fruit)	70.54 ± 11.11aA	68.48 ± 4.28aA	75.79 ± 8.00aA	38.66 ± 3.83bB

WG1 and SK1, whole grapes and skins of BRS Carmem from harvest 1, respectively; WG2 and SK2 whole grapes and skins of BRS Carmem from harvest 2, respectively; ^1^ Assignation: M, myricetin; Q, quercetin; L, laricitrin; S, syringetin; I, isorhamnetin; glcU, 3-glucuronide; gal, 3-galactoside; glc, 3-glucoside; cmglc, coumaroyl; ND, not detected; a, b: different letters in the same row indicate significant differences (α = 0.05) between the whole grapes and the skins of each harvest. A, B: different letters in the same row indicate significant differences (α = 0.05) between the whole grapes or the skins of both harvests. Adapted from Nishiyama, 2020 [32].

**Table 3:** Anthocyanins in whole BRS Carmem grape according to HPLC-DAD-ESI-MS/MS (positive ionization mode), molar profiles (percentage of each individual anthocyanins regarding the total content) and total concentration given as mean values ± standard deviation (*n* = 2).

Anthocyanin ^1^	% Molar Porcentages ^2^	
	WG1	WG2
dp-3,5-diglc	25.11 ± 2.94A	0.48 ± 0.15B
cy-3,5-diglc	1.48 ± 0.09A	0.05 ± 0.02B
pt-3,5-diglc	13.11 ± 0.87A	0.97 ± 0.28B
pn-3,5-diglc	3.09 ± 0.75A	1.73 ± 0.24A
mv-3,5-diglc	14.10 ± 1.50A	15.14 ± 2.31A
pt-3-acglc-5-glc	ND	0.25 ± 0.04
pn-3-acglc-5-glc	0.18 ± 0.03B	0.37 ± 0.05A
mv-3-acglc-5-glc	0.78 ± 0.20B	2.55 ± 0.17A
dp-3-cmglc-5-glc	25.81 ± 1.59A	6.98 ± 0.39B
cy-3-cmglc-5-glc	1.84 ± 0.38A	0.43 ± 0.12B
pt-3-cmglc-5-glc	4.70 ± 0.48B	13.03 ± 0.91A
pn-3-cmglc-5-glc	1.62 ± 0.45B	3.34 ± 0.25A
mv-3-cmglc-5-glc	7.79 ± 1.70B	52.49 ± 4.34A
dp-3-cfglc-5-glc	0.38 ± 0.06	ND
cy-3-cfglc-5-glc	ND	0.01 ± 0.00
pt-3-cfglc-5-glc	ND	0.57 ± 0.04
pn-3-cfglc-5-glc	ND	0.42 ± 0.19
mv-3-cfglc-5-glc	ND	1.21 ± 0.08
dp-3-glc	30.14 ± 1.49A	4.05 ± 1.25B
cy-3-glc	3.86 ± 0.18A	0.70 ± 0.14B
pt-3-glc	6.67 ± 1.04A	2.31 ± 0.24B
pn-3-glc	1.94 ± 0.62A	2.58 ± 0.27A
mv-3-glc	3.91 ± 0.73A	5.66 ± 0.92A
dp-3-acglc	2.32 ± 0.41A	1.85 ± 0.40A
pt-3-acglc	1.40 ± 0.19A	1.29 ± 0.02A
pn-3-acglc	ND	2.74 ± 0.31
mv-3-acglc	0.94 ± 0.22B	2.54 ± 0.27A
dp-3-cmglc	39.83 ± 2.69A	27.76 ± 0.02B
cy-3-cmglc	1.92 ± 0.14	ND
pt-3-cmglc	4.44 ± 0.15B	19.12 ± 0.90A
pn-3-cmglc	0.64 ± 0.15B	2.28 ± 0.25A
mv-3-cmglc	1.98 ± 0.36B	25.65 ± 2.25A
mv-3-cfglc	ND	1.46 ± 0.22
Ratio 3,5-diglc/3-glc	2.22 ± 0.16B	5.64 ± 0.23A
mg/kg fruit (mv-3,5-diglc)	1650.19 ± 110.65A	1631.41 ± 127.37A
mg/kg fruit (mv-3-glc)	559.49 ± 2.92A	194.51 ± 23.18B
Total mg/kg fruit (mv-3,5-diglc)	2528.01 ± 106.08A	1921.58 ± 161.95B

WG1 and WG2, whole grapes of BRS Carmem from harvest 1 and harvest 2, respectively; ^1^Assignation: dp, delphinidin; cy, cyanidin; pt, petunidin; pn, peonidin; mv, malvidin; 3,5-diglc, 3,5-diglucosides; 3-acglc-5-glc, 3-(6″-acetyl)-glucoside-5-glucoside; 3-cmglc-5-glc, 3-(6″-*p*-coumaroyl)-glucoside-5-glucoside; 3-cfglc-5-glc, 3-(6″-*p*-caffeoyl)-glucoside-5-glucoside; 3-glc, 3-glucoside; 3-acglc, 3-(6″-acetyl)-glucoside; ^2^, % molar percentage of each group (mono- and di-glucosides) totalizes 100%; A, B: different letters in the same row indicate significant differences (α = 0.05) between the harvests. Adapted from Nishiyama, 2020 [32].

**Table 4:** Flavan-3-ol (monomers and dimers), proanthocyanidins (terminal and extension unit) in skins and seeds of BRS Carmem according to HPLC-ESI-MS/MS-MRM, the concentration of each individual flavan-3-ol, molar profiles, and the structural characterization of proanthocyanidins and total concentrations given as mean values ± standard deviation (*n* = 2 for SK1, SE1 and SE2 and, *n* = 3 for SK2).

Compound ^1^	mg/kg Fruit ^2^			
	SE1	SK1	SE2	SK2
C	56.10 ± 0.79aB	0.62 ± 0.11bA	84.24 ± 1.21aA	0.65 ± 0.04bA
EC	18.66 ± 0.92aB	0.15 ± 0.00bA	40.50 ± 3.72aA	0.11 ± 0.01bB
GC	0.06 ± 0.00b	0.19 ± 0.02aA	ND	0.16 ± 0.04A
EGC	0.21 ± 0.04a	0.21 ± 0.01aA	ND	0.12 ± 0.01B
CG	0.09 ± 0.00B	ND	0.13 ± 0.00A	ND
ECG	0.81 ± 0.01aA	0.02 ± 0.02bA	1.15 ± 0.11aA	0.04 ± 0.00bA
MG1	ND	ND	1.97 ± 0.35	ND
MG2	ND	ND	0.55 ± 0.01	ND
Total monomers ^3^	179.05 ± 18.29aA	1.17 ± 0.15bA	127.18 ± 5.08aB	1.04 ± 0.07bA
PB1	7.04 ± 1.12aB	0.08 ± 0.01bA	17.59 ± 0.83aA	2.93 ± 0.25bA
PB2	1.12 ± 0.06aB	0.08 ± 0.01bB	35.41 ± 0.15aA	0.42 ± 0.08bA
PB4	12.23 ± 0.38aA	0.18 ± 0.01bA	2.40 ± 0.02aB	0.16 ± 0.03bA
Total dimers ^3^	24.15 ± 0.20aB	0.73 ± 0.01bB	55.54 ± 0.71aA	3.52 ± 0.35bA
Total ^3^	203.20 ± 18.49aA	1.90 ± 0.16bB	182.71 ± 4.37aA	4.56 ± 0.36bA
PA ^3^	453.57 ± 79.99aA	37.58 ± 0.04bB	299.86 ± 10.82aA	98.92 ± 9.18bA
mDP	3.06 ± 0.19bA	5.93 ± 0.21aA	3.64 ± 0.36bA	5.65 ± 0.07aA
% galloylation	8.71 ± 0.86aA	1.95 ± 0.12bB	7.25 ± 0.29aA	3.95 ± 0.07bA
% prodelphinidin	0.81 ± 0.06bA	25.02 ± 0.93aA	0.46 ± 0.08bB	16.91 ± 1.68aB
% C-term	48.13 ± 7.33aA	60.28 ± 1.14aA	64.68 ± 1.78aA	62.06 ± 2.62aA
% EC-term	41.56 ± 5.58aA	11.56 ± 0.24bA	31.04 ± 1.55aA	11.04 ± 1.36bA
% GC-term	0.18 ± 0.12b	13.40 ± 0.29aA	ND	13.99 ± 2.58A
% EGC-term	0.37 ± 0.04b	13.21 ± 0.44aA	ND	10.59 ± 0.79B
% CG-term	0.04 ± 0.01A	ND	0.06 ± 0.00A	ND
% ECG-term	9.73 ± 1.81aA	1.54 ± 0.17bB	0.56 ± 0.08bB	2.33 ± 0.02aA
% MG-term	ND	ND	3.65 ± 0.30	ND
% C-ext	5.01 ± 5.00aA	0.58 ± 0.1aB	10.49 ± 0.80aA	3.90 ± 0.36bA
% EC-ext	86.95 ± 5.61aA	73.98 ± 1.39bA	78.87 ± 0.69aA	70.77 ± 2.38bA
% GC-ext	0.05 ± 0.00b	0.47 ± 0.08a	ND	ND
% EGC-ext	0.56 ± 0.04bA	22.97 ± 1.22aA	0.64 ± 0.09bA	20.53 ± 2.02aA
% CG-ext	7.43 ± 0.33aB	1.99 ± 0.15bB	10.00 ± 0.03aA	4.80 ± 0.09bA

SE1 and SK1, seeds and skins of BRS Carmem from harvest 1, respectively; WG2 and SK2, whole grapes and skins of BRS Carmem from harvest 2, respectively; ND, not detected; ^1^ Assignation: C, catechin; EC, epicatechin; GC, gallocatechin; EGC, epigallocatechin; CG, catechin gallate; ECG, epicatechin gallate; PB1, proanthocyanidin B1; PB2, proanthocyanidin B2; PB4, proanthocyanidin B4; PA, total proanthocyanidins; mDP, mean degree of polymerization; MG, monoglucoside; MG1, monoglucoside 1; MG2, monoglucoside 2; -term, terminal unit; -ext, extension unit; -term and -ext separated totaling 100% for each one; ^2^, expressed in mg/kg fruit; ^3^, totals expressed as catechin equivalents in mg/kg fruit; a, b: different letters in the same row indicate significant differences (α = 0.05) between the whole grapes and the skins of each harvest. A, B: different letters in the same row indicate significant differences (α = 0.05) between the whole grapes or the skins of both harvests. Adapted from Nishiyama, 2020 [32].

**Table 5:** Hydroxycinnamic acid derivatives (HCAD) in whole grapes and skins of BRS Carmem grape according to HPLC-DAD-ESI-MS/MS (negative ionization mode), molar profiles (percentage of each individual HCAD regarding the total content) and total concentration given as mean values ± standard deviation (*n* = 3).

HCAD	% Molar Percentage			
	WG1	SK1	WG2	SK2
*trans-*caftaric	53.36 ± 2.015aA	50.76 ± 3.41aA	24.77 ± 5.31aB	33.56 ± 4.55aB
*trans-*cutaric	18.94 ± 2.00aA	21.27 ± 5.46aA	13.69 ± 5.60aA	11.74 ± 0.90aB
1-glc-cumaric	14.61 ± 1.29aA	6.96 ± 5.20aA	16.95 ± 1.44aA	13.63 ± 1.03bA
2-glc-cumaric	6.16 ± 0.60aB	8.26 ± 2.06aA	14.63 ± 1.16aA	12.80 ± 4.46aA
*trans-*fertaric	1.89 ± 0.33bB	7.05 ± 0.48aA	8.88 ± 0.83aA	9.47 ± 4.70aA
3-glc-cumaric	5.04 ± 1.07aB	5.70 ± 1.11aB	21.08 ± 0.67aA	18.80 ± 3.28aA
Total ^1^	383.98 ± 32.72aA	173.95 ± 91.36bA	67.09 ± 21.69aB	21.74 ± 4.00bB

WG1 and SK1, whole grapes and skins of BRS Carmem from harvest 1, respectively; WG2 and SK2, whole grapes and skins of BRS Carmem from harvest 2, respectively; ^1^, total expressed in mg of caftaric acid/kg fruit; a, b: different letters in the same row indicate significant differences (α = 0.05) between the whole grapes and the skins of each harvest. A, B: different letters in the same row indicate significant differences (α = 0.05) between the whole grapes or the skins of both harvests. Adapted from Nishiyama, 2020 [32].

**Table 6:** Stilbenes in skins and seeds of BRS Carmem grape according to HPLC-ESI-MS/MS-MRM, molar profiles (percentage of each individual stilbenes) and total concentration given as mean values ± standard deviation (*n* = 2 for SK1, SE1 and SE2 and, *n* = 3 for SK2).

Stilbenes	% Molar Percentage			
	SE1	SK1	SE2	SK2
*trans*-piceid	98.73 ± 0.71aA	78.79 ± 3.20bA	1.94 ± 0.26bB	29.88 ± 6.73aB
*cis*-piceid	1.27 ± 0.71bB	21.21 ± 3.20aA	6.95 ± 0.36aA	4.96 ± 1.51aA
*cis*-resveratrol	ND	ND	91.11 ± 0.10a	65.16 ± 8.06b
Total ^1^	48.67 ± 4.76aB	21.87 ± 4.44bA	896.25 ± 265.34aA	3.69 ± 0.45bB

SE1 and SK1, seeds and skins of BRS Carmem from harvest 1, respectively; WG2 and SK2, whole grapes and skins of BRS Carmem from harvest 2, respectively; ND, not detected; ^1^, total expressed in µg 3-glc-resveratrol/kg fruit. a, b: different letters in the same row indicate significant differences (α = 0.05) between the whole grapes and the skins of each harvest. A, B: different letters in the same row indicate significant differences (α = 0.05) between the whole grapes or the skins of both harvests. Adapted from Nishiyama, 2020 [32].

## Data Availability

The data presented in this study are available on request from the corresponding author.

## Supplementary Materials

The following supporting information can be downloaded at: https://www.mdpi.com/article/10.3390/foods12132608/s1, Table S1: Chromatographic and spectroscopic characteristics of the flavonols, anthocyanins and hydroxycinnamic acid derivatives identified in BRS Carmem grapes by HPLC-DAD-ESI-MS/MS.
