# Supplementary material for: Phenolic Composition of Brazilian BRS Carmem (Muscat Belly A × BRS Rúbea) Grapes: Evaluation of Their Potential Use as Bioingredients
Source: Foods. 2023 Jul 5;12(13):2608. doi: 10.3390/foods12132608 (PMC10341059; doi:10.3390/foods12132608)
Supplement: Supplementary file 1 [file foods-12-02608-s001.zip › foods-2448122-SI.pdf]

Table S1. Chromatographic and spectroscopic characteristics of the flavonols, anthocyanins and hydroxycinnamic acid derivatives identified in BRS Carmem grapes by HPLC-DAD-ESI-MS/MS.

Phenolic compound	Compound	Retention Time (min)	Molecular ion; product ions (m/z)
Flavonol (negative ionization mode)	M-glcU	16.4	493; 317
	M-glc	18.3	479; 317
	Q-gal	25.4	463; 301
	Q-glcU	25.9	477; 301
	Q-glc	27.4	463; 301
	L-glc	30.5	493; 331
	S-gal	37.1	507; 345
	I-glc	37.3	477; 315
	S-glc	39.1	507; 345
	S-cmglc	52.4	609, 345
	M free	-	317; 317
	Q free	-	301; 301
	L free	-	331; 331
	I free	-	315; 315
	S free	-	345; 345
Anthocyanin (positive ionization mode)	dp-3,5-diglc	6.3	627; 465, 303
	cy-3,5-diglc	9.7	611; 449, 287
	dp-3-glc	10.4	465;303
	pt-3,5-diglc	12.0	641; 479, 317
	cy-3-glc	13.3	449;287
	pn-3,5-diglc	14.5	625; 463, 301
	pt-3-glc	15.7	479; 317
	mv-3,5-diglc	16.1	655; 493, 331
	pn-3-glc	18.4	463; 301
	pt-3-acglc-5glc	19.5	683; 521,317
	mv-3-glc	20.1	493; 331
	dp-3-cfglc-5-glc	20.2	789; 627, 465, 303
	dp-3-acglc	21.4	507; 465,303
	pn-3-acglc-5glc	22.1	667; 505, 463, 301
	mv-3-acglc-5glc	23.0	697; 535, 493, 331
	cy-3-cfglc-5-glc	23.5	773; 611, 287
	dp-3-cmglc-5-glc	24.5	773; 611, 465, 303
	pt-3-cfglc-5-glc	24.8	803; 641, 317
	pt-3-acglc	25.9	521; 479, 317
	cy-3-cmglc-5-glc	26.9	757; 595, 287
	pn-3-cfglc-5-glc	27.0	787; 625, 301
	mv-3-cfglc-5-glc	27.9	817; 655, 331
	pt-3-cmglc-5-glc	28.1	787; 625,317
	dp-3-cmglc	28.5	611; 303
	pn-3-acglc	28.9	505; 301
	cy-3-cmglc	29.8	595; 287
	mv-3-acglc	30.3	535; 331
	pn-3-cmglc-5-glc	30.7	771; 609, 307
	mv-3-cmglc-5-glc	31.4	801; 639, 331
	mv-3-cfglc	32.1	655; 331
	pt-3-cmglc	32.8	625; 317
	pn-3-cmglc	35.9	609; 301
	mv-3-cmglc	37.0	639; 331
Hydroxycinnamic acid derivatives (negative ionization mode)	trans-caftaric acid	3.7	311; 179, 149, 135
	trans-cutaric acid	5.2	325; 193, 149, 119
	1-glc-cumaric acid	6.9	325; 163, 145
	2-glc-cumaric acid	7.4	325; 163, 145
	trans-fertaric acid	7.8	325; 193, 149
	3-glc-cumaric acid	8.7	325; 163, 145
